# 1,8-Cineol Attenuates Checkpoint Molecule PDL-1 and Adhesion Molecule CX3CR1 in Circulating Monocytes in Otitis Media Patients

**DOI:** 10.3390/jpm14030279

**Published:** 2024-03-01

**Authors:** Anke Leichtle, Stephanie Jeschke, Kirstin Plötze-Martin, Christian Idel, Karl-Ludwig Bruchhage, Ralph Pries

**Affiliations:** Department of Otorhinolaryngology, University of Lübeck, 23538 Lübeck, Germany; anke.leichtle@uksh.de (A.L.); stephanie.jeschke@uksh.de (S.J.); kirstin.ploetze-martin@uksh.de (K.P.-M.); christian.idel@uksh.de (C.I.); karl-ludwig.bruchhage@uksh.de (K.-L.B.)

**Keywords:** chronic otitis media, 1,8-Cineol, monocytes, adhesion molecules, PD-L1, CXCL10

## Abstract

Background: Peripheral blood monocytes can be subdivided into different subsets based on the CD14/CD16 surface characteristics. Monocytes are a major source of cytokine secretion of pro-inflammatory immune responses, whereas CD16^+^ monocyte subsets can also contribute to persistent inflammation in the context of chronic diseases. However, the regulation and cellular characteristics of circulating monocyte subsets in patients with chronic otitis media (COM), one of the largest public health burdens, remains largely unknown. Materials and Methods: In this study, we analyzed individual distributions of circulating monocyte subsets and associated protein expression levels of adhesion protein and chemokine receptors CD11a (integrin-α L; LFA-1), CD11b (integrin-α M; Mac-1), and CD11c (integrin-α X), CX3CR1 (CX3CL1 receptor), as well as checkpoint molecule PD-L1 (programmed cell death ligand-1), in a gender-balanced cohort of 14 patients with chronic otitis media using flow cytometry, especially in view of the therapeutic impact of the natural plant-derived monoterpene oxide 1,8-Cineol. Furthermore, using the human monocyte cell line THP-1 as a model, we investigated the influence of anti-inflammatory 1,8-Cineol on monocytic cytokine secretion patterns using human cytokine arrays and ELISA measurements. Results: The data revealed significantly elevated expression levels of all analyzed adhesion molecules in certain monocyte subsets in COM patients; CX3CR1 was especially significantly down-regulated in response to 1,8-Cineol administration. Moreover, the data revealed significantly increased monocytic PD-L1 expression levels in circulating classical and intermediate monocyte subsets from COM patients compared to healthy donors, but also a significant decrease in PD-L1 in intermediate monocytes upon 1,8-Cineol therapy compared to the pre-treatment situation. Furthermore, the increased secretion of cytokine CXCL10 by THP-1 monocytes in response to LPS was found to be strongly attenuated by 1,8-Cineol. Plasma levels of CXCL10 were also significantly increased in COM patients, but no significant differences between the pre and post 1,8-Cineol situation were observed. Conclusions: The present study revealed new insights into the bioactive anti-inflammatory effects of 1,8-Cineol in terms of monocyte adhesion and immune regulation. Our data suggest the potential role of cytokine CXCL10 in COM development and maintenance, which is also involved in the activity of its concomitant disease, rheumatoid arthritis.

## 1. Introduction

Monocytes are important mediators of the inflammatory immune response and a major source of serum cytokines. Inflammation-associated environmental signals mediate rapid monocyte recruitment, and participate in the clearance of pathogens and the initiation of further adaptive immune responses [1]. Moreover, inflammatory monocytes and macrophages can also contribute to persistent inflammation in the context of chronic diseases such as atherosclerosis or chronic otitis media (COM) [2,3]. COM is one of the most common public health burdens worldwide, resulting in hearing loss as well as concomitant life-threatening central nervous system diseases [4]. Moreover, COM-related inflammation is associated with an increased permeability of the cochlear membranes that face the middle ear cavity, allowing bacteria to enter the inner ear [5,6] and resulting in partial or total irreversible deafness. Further COM-related otogenic complications, such as mastoiditis, labyrinthitis, thrombosis of the venous sinus or intracranial complications, may occur without clinical treatment [7]. 

In everyday life, patients with COM suffer from hearing impairment, difficulties in conversations, and social distancing. Without sufficient therapy, there is no way out of this descending spiral. This is especially the case in therapy-persistent COM due to antimicrobial resistance; up-to-date, middle ear surgery, with the complete removal of the inflammation foci and appropriate surgical rehabilitation, including the reconstruction of the tympanic membrane, the classic hearing structures or implantable hearing devices, such as an active middle ear implant (VSB), is the only way to treat this disease [8].

COM is connected with different inflammatory processes within the middle ear [9,10], whereas infections with Gram-negative bacteria, such as *Pseudomonas aeruginosa*, are the main cause of COM development [8,11]. In this context, lipopolysaccharide (LPS) is a major component of the cellular envelope of Gram-negative bacteria and it has been shown in mice that LPS-induced COM leads to different inflammatory reactions, such as the infiltration of macrophages and expression of inflammatory cytokines [12]. 

However, a detailed understanding of the abundances of peripheral blood monocyte subsets and regulation patterns of cytokines and proteins required for adhesion, as well as of the invasion and immune regulation in patients with chronic otitis media, remains incomplete. Monocytes can be subdivided into three different subsets in view of their CD14 and CD16 cell surface expression levels: “classical” monocytes (CD14^++^CD16^−^), “intermediate” monocytes (CD14^+^CD16^+^), and “non-classical” monocytes (CD14^dim+^CD16^+^) [13,14]. 

The aim of this study was to analyze the individual distributions of circulating monocyte subsets in COM patients as well as the associated expression patterns of proteins required for adhesion and chemokine recognition, such as CD11a (integrin-α L; LFA-1), CD11b (integrin-α M; Mac-1), CD11c (integrin-α X), CX3CR1 (CX3CL1 receptor), and checkpoint molecule PD-L1 (programmed cell death ligand-1), using flow cytometry, all of which are known to be differentially expressed in response to different environmental conditions [15] and in correlation with the clinical situation. 

Moreover, all of these investigations were performed before and after the application of the natural plant-derived monoterpene oxide 1,8-Cineol, which is the active component of the clinically approved drug Soledum^®^ (Cassella-med GmbH & Co. KG, Cologne, Germany), which is applied to treat different human diseases such as chronic and acute airway inflammations such as bronchitis, chronic rhinosinusitis or chronic obstructive pulmonary disease (COPD) [16,17]. 1,8-Cineol is the major bacteriostatic agent of several species of the genus eucalypt, although details of the underlying mechanisms still remain unclear. Strong antimicrobial effects of 1,8-Cineol have been observed to inhibit methicillin-resistant *S. aureus* strains in co-administration with chlorhexidine gluconate, and also *E. coli*, *K. pneumoniae*, *E. faecalis*, and *C. albicans* strains with a weaker effect [18]. Furthermore, 1,8-Cineol containing essential oils from the leaf parts of *E. globulus* also revealed significant anti-bacterial properties against *S. aureus* in agar diffusion assays [19].

Earlier studies revealed that 1,8-Cineol attenuates different pro-inflammatory cytokines, such as TNF-α, IL-1β, and IL-6, from monocytes [20]. Moreover, 1,8-Cineol revealed the potential to significantly inhibit different biosynthetic signaling cascades such as the WNT/ß-catenin or the NFκB signaling pathways [21,22]. However, the extensive influence of 1,8-Cineol on the overall expression patterns of monocytic inflammatory cytokines and chemokines have been explored only to a small extent so far. Therefore, using the THP-1 cell line as a model, we comprehensively evaluated the influence of 1,8-Cineol on the secretion levels of 105 different cytokines and chemokines in response to bacterial LPS stimulation. The study aimed to increase our understanding of chronic inflammation-driven immunological changes in monocytic cells and the possible therapeutic improvement via 1,8-Cineol administration at a cellular level in chronic otitis media. 

## 2. Materials and Methods

### 2.1. Ethics Statement and Characteristics of Examined Patients

Patients with otitis media were examined at the Department of Otorhinolaryngology, University Hospital Schleswig-Holstein, Campus Lübeck, after signing a written declaration of consent. The study was approved by the ethics committee of the University of Lübeck (approval number 21–183) and was carried out in harmony with the ethical principles of the WMA Declaration of Helsinki. All enrolled patients (*n* = 14) in this study suffered from rhinosinusitis and persistent COM, both of which share the same respiratory epithelium. COM patients underwent antibiotic therapy and middle ear surgery and developed microbial resistances against many or all available classical antibiotics. Often, there were no therapeutic options left and, therefore, we treated them with 1,8-Cineol. 1,8-Cineol (CNL-1976) was used in terms of the clinically approved drug Soledum^®^ Kapseln forte (capsules) (Cassella-med GmbH & Co. KG, Cologne, Germany, also available as Soledum addicur capsules registered in Germany since 1 November 2019), which is authorized in various European countries and commonly applied to treat a broad range of human diseases such as chronic and acute airway inflammations [17,23,24,25]. 

As part of the therapeutic treatment, Soledum capsules forte (3 × 200 mg Cineol/day) were orally administered to the patients over 14 days. They did not receive any local or systemic antibiotic treatments during this time period. For clinical diagnostics, we examined the change in pain, hearing loss, otorrhea, pressure in the tuba auditive, and other factors related to quality of life. 

### 2.2. Blood Collection and FACS Analysis of Monocyte Subsets in Whole Blood

Blood samples were obtained from healthy donors (*n* = 10) and patients with chronic otitis media (*n* = 14; mean age of 58; 6 female/ 8 male) before and after 14 days of 1,8-Cineol administration. Whole blood samples were taken by venipuncture into a sodium citrate containing S-Monovettes (Sarstedt; Nümbrecht, Germany). Within 4 h after blood sampling, 20 µL of citrate blood was diluted with 80 µL phosphate-buffered saline (PBS). Cells were stained for FACS analysis with the following antibodies: CD45-PE, CD14-FITC, CD16-BV-510, HLA-DR-APC-Cy7, CX3CR1-BV421, CD11a-PE-Cy7, CD11b-PerCP, CD11c-BV421, and PD-L1-APC (all from Biolegend, San Diego, CA, USA) for 25 min in the dark. Subsequently, 650 µL RBC Lysis Buffer (Biolegend) were added to the samples and, after another 20 min of incubation, the suspension was centrifuged at 400× *g* for 5 min and the supernatant was discarded. Cell sediment was resuspended in 100 µL fresh PBS and used for FACS analysis. Flow cytometry was carried out with a MACSQuant 10 flow cytometer (Miltenyi Biotec, Bergisch-Gladbach, Germany) and data analyses were performed using FlowJo^TM^ software version 10.0 (FlowJo, LLC, Ashland, OR, USA). 

### 2.3. THP-1 Cells and Culture Conditions

In vitro cell culture experiments were performed culturing the non-adherent monocyte cell line THP-1 in RPMI 1640 medium supplemented with 10% heat-inactivated fetal bovine serum (FBS), 1% sodium pyruvate, and 1% streptomycin/penicillin at 37 °C and 5% CO_2_ under a humidified atmosphere. Cells were subcultured every 72 h at a maximum density of 1 × 10^6^ cells/mL. THP-1 cells were incubated with 10 ng/mL lipopolysaccharide (LPS) (Sigma Aldrich, St. Louis, MO, USA) and 1,8-Cineol (Cassella-med GmbH & Co. KG, Cologne, Germany) at a concentration of 1 mM for 24 h. Additionally, Propidium iodide (PI) and Annexin V-APC staining was performed to determine cellular viability. Flow cytometric investigations were performed using a MACSQuant 10 flow cytometer (Miltenyi Biotec, Bergisch-Gladbach, Germany) and data were evaluated using the FlowJo software version 10.0 (FlowJo, LLC, Ashland, OR, USA). 

### 2.4. Cytokine Analyses

To determine THP-1 cytokine expression patterns in response to lipopolysaccharide (LPS) from *Escherichia coli* in the presence and absence of 1,8-Cineol, cytokine arrays were performed. Cell culture supernatants were removed after incubation and instantly frozen with liquid nitrogen and preserved at −80 °C. Proteome Profiler^TM^ Human XL cytokine arrays (R&D Systems, Minneapolis, MN, USA) were hybridized with the cell culture supernatants as specified by the supplier. Expression was visualized using an enhanced chemiluminescence detection kit (R&D Systems, Minneapolis, MN, USA). Semiquantitative analysis was performed by measuring the density of the bands using an iBright CL 1000 biomolecular imager (Invitrogen, Carlsbad, CA, USA). The protein concentrations of the human CXCL10 were determined according to the protocol given by the commercial ELISA assay (R&D Systems, USA).

### 2.5. Statistical Analyses

GraphPad Prism Version 7.0f Statistical was used to evaluate the collected data. The mean and standard error (SEM) were shown and differences between groups were determined after testing for Gaussian distribution (normality tests) and applying parametric (Student’s *t*-Test) or non-parametric 1-way ANOVA with Bonferroni post hoc test. The correlation between parameters was calculated using multivariate regression with the Pearson correlation coefficient. *p* < 0.05 (*), *p* < 0.01 (**), *p* < 0.001 (***). Further statistical information is given in the respective figure legends as appropriate.

## 3. Results

### 3.1. Monocyte Subset Characteristics upon 1,8-Cineol Tretament of Otitis Media Patients

We analyzed the percentages of CD14^++^CD16^−^ (classical), CD14^++^CD16^+^ (intermediate), and CD14^dim+^CD16^+^ (non-classical) monocyte subsets in the peripheral blood of COM patients (*n* = 14) prior to and 14 days after 1,8-Cineol administration, and compared the results with healthy donors (*n* = 10). The gating of flow cytometric measurements of CD14- and CD16-defined monocyte subsets was carried out according previously published methods [26]. 

**Figure 1:**
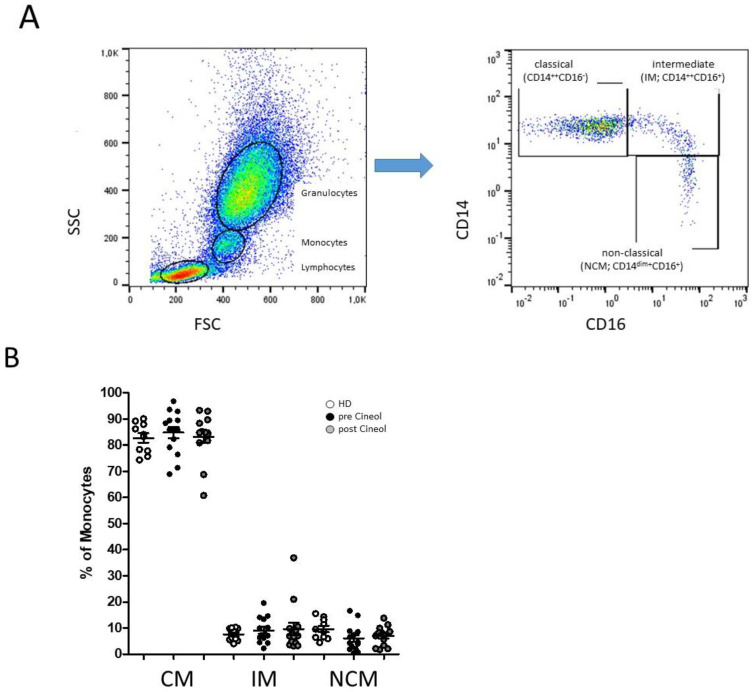
Flow cytometric analysis of CD14- and CD16-characterized monocyte subsets. (**A**): Representative example gating scheme of peripheral monocyte subset analysis by flow cytometry. (**B**): Whole blood analysis revealed similar median abundances of classical monocytes (CM; CD14^++^CD16−), intermediate (IM; CD14^++^CD16^+^), and non-classical monocytes (NCM; CD14^dim+^CD16^+^) in patients with chronic otitis media (COM) before and after 1,8-Cineol administration compared to healthy donors.

Most patients revealed a normal distribution of blood monocyte subsets, whereas two COM patients revealed decreased abundances of classical monocytes accompanied with increased levels of CD16* monocytes. Overall, the data revealed no significant differences between monocyte subsets from healthy donors and COM patients before and after 1,8-Cineol treatment (Figure 1).

Moreover, the protein expression patterns of adhesion molecules and chemokine receptors CD11a (integrin-α L; LFA-1), CD11b (integrin-α M; Mac-1), CD11c (integrin-α X), and CX3CR1 (CX3CL1 receptor) on CD14/CD16 monocyte subsets were analyzed before and after 1,8-Cineol administration using flow cytometry. 

**Figure 2:**
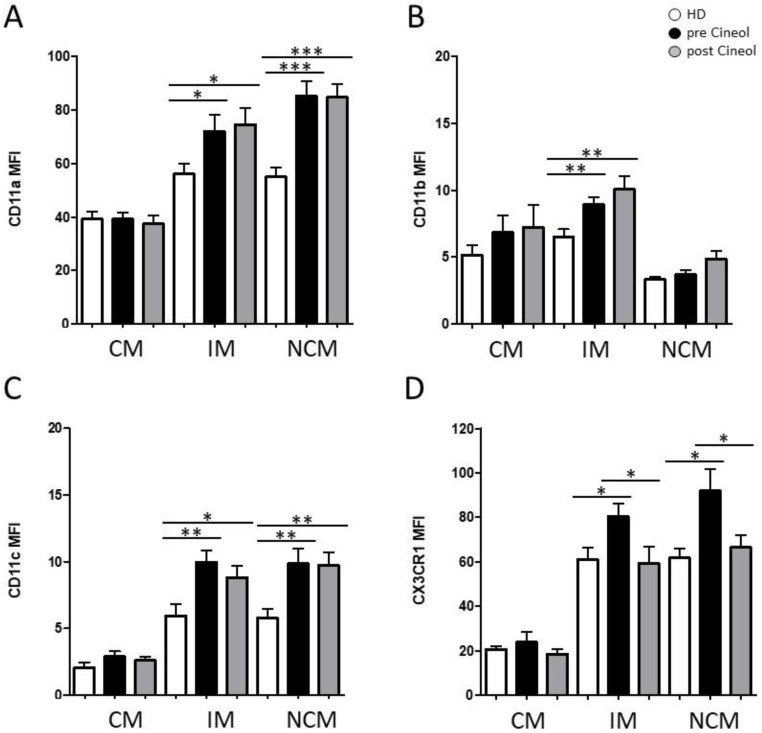
Flow cytometric analysis of monocytic adhesion molecules: (**A**) CD11a, (**B**) CD11b, (**C**) CD11c, and (**D**) CX3CR1 on classical (CM; CD14^++^CD16^−^), intermediate (IM; CD14^++^CD16^+^), and non-classical (NCM; CD14^dim+^CD16^+^) monocytes from COM patients before (pre) and after (post) 1,8-Cineol administration compared to healthy donors (HD). *: *p* < 0.05; **: *p* < 0.01; ***: *p* < 0.001. MFI: mean fluorescence intensity.

The expression of CD11a was significantly higher in intermediate monocytes (pre: *p* = 0.0400; post: *p* = 0.0174) and non-classical monocytes (pre: *p* ≤ 0.0001; post: *p* ≤ 0.0001) in COM patients compared to healthy donors, with no significant differences between the pre versus post situations (Figure 2A). The expression of CD11b was significantly higher in intermediate monocytes (pre: *p* ≤ 0.0084; post: *p* ≤ 0.0082) compared to healthy donors, also with no significant influence after 1,8-Cineol (Figure 2B).

Furthermore, measurements revealed a significantly increased expression of CD11c in intermediate monocytes (pre: *p* = 0.0053; post: *p* = 0.0314) and non-classical monocytes (pre: *p* = 0.0054; post: *p* = 0.0033) in COM patients compared to healthy donors (Figure 2C).

Notably, we observed a significantly increased expression of adhesion molecule CX3CR1 on intermediate monocytes (pre: *p* = 0.0254) and non-classical monocytes (pre: *p* = 0.0254) in COM patients compared to healthy donors, which were significantly decreased in response to 14 days of 1,8-Cineol administration (IM: *p* = 0.0468; NCM: *p* = 0.0393) (Figure 2D).

**Figure 3:**
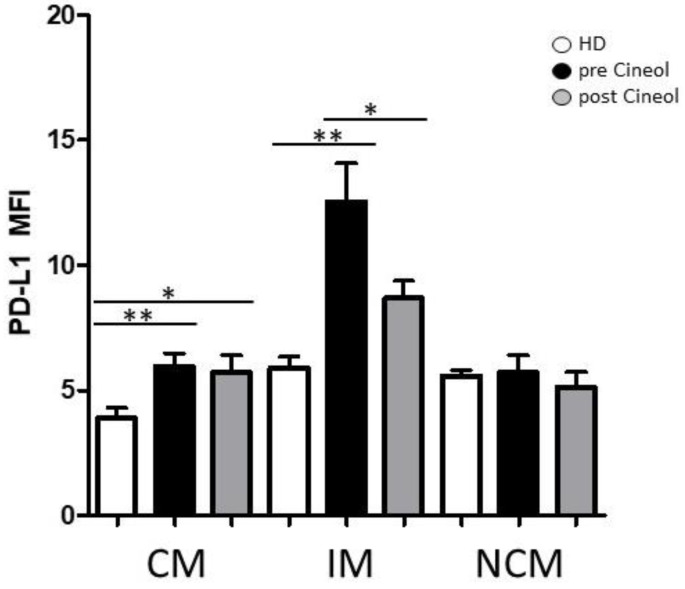
Flow cytometric analysis of checkpoint molecule PD-L1. PD-L1 expression on CD14/CD16 monocyte subsets (CM: classical monocytes; IM: intermediate monocytes; NCM: non-classical monocytes) from COM patients was measured before and after 1,8-Cineol administration and compared to healthy donors (HD). *: *p* < 0.05; **: *p* < 0.01. MFI: mean fluorescence intensity.

In addition, expression levels of checkpoint molecule PD-L1 were analysed in monocyte subsets before and after 1,8-Cineol therapy and these were compared to healthy donors. Our data revealed significantly increased monocytic PD-L1 expression levels in circulating classical and intermediate monocyte subsets from COM patients compared to healthy donors, but also a significant decrease in PD-L1 in intermediate monocytes upon 1,8-Cineol therapy compared to the pre-treatment situation (Figure 3).

### 3.2. Treatment of THP-1 Monocytes with Lipopolysaccharide and 1,8-Cineol

**Figure 4:**
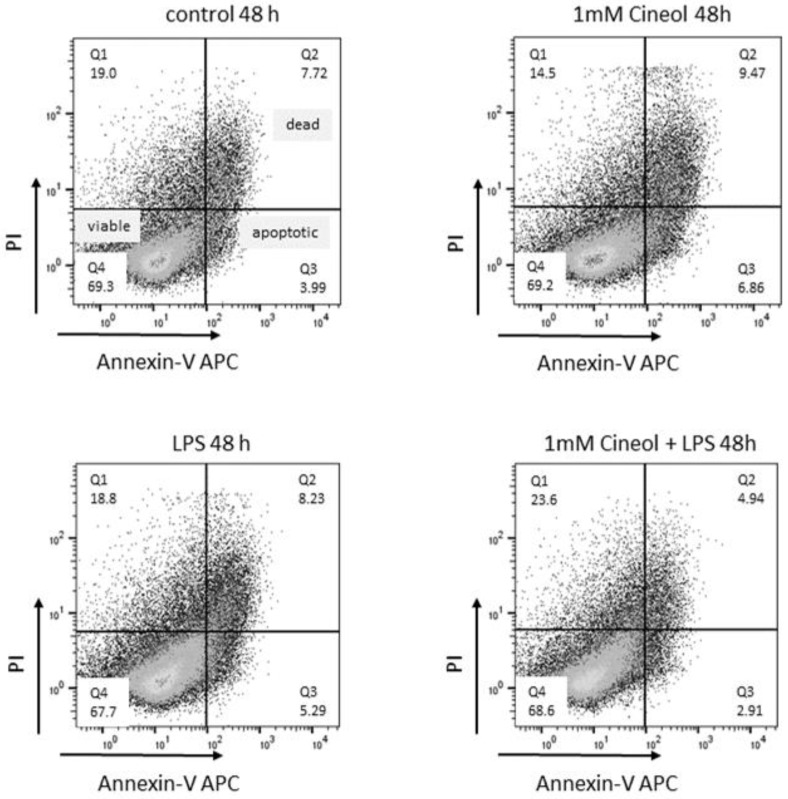
Viability and apoptosis induction in response to LPS and/or 1,8-Cineol treatment of THP-1 monocytes. Representative examples of flow cytometric analyses of Propidium iodide (PI) and Annexin V-APC revealed no cytotoxic effects compared to the medium control.

Cells of the human monocyte leukaemia cell line THP-1 were treated with 10 ng/mL lipopolysaccharide (LPS) and/or 1 mM 1,8-Cineol for 24 h. Propidium iodide (PI) and Annexin V-APC staining revealed no cytotoxic effects on the cellular viability (Figure 4).

To determine the impact of 1,8-Cineol on LPS-induced monocytic cytokine expression patterns, the expression levels of 105 different cytokines and chemokines in supernatants of the treated monocyte cell cultures were screened using a human cytokine antibody array.

**Figure 5:**
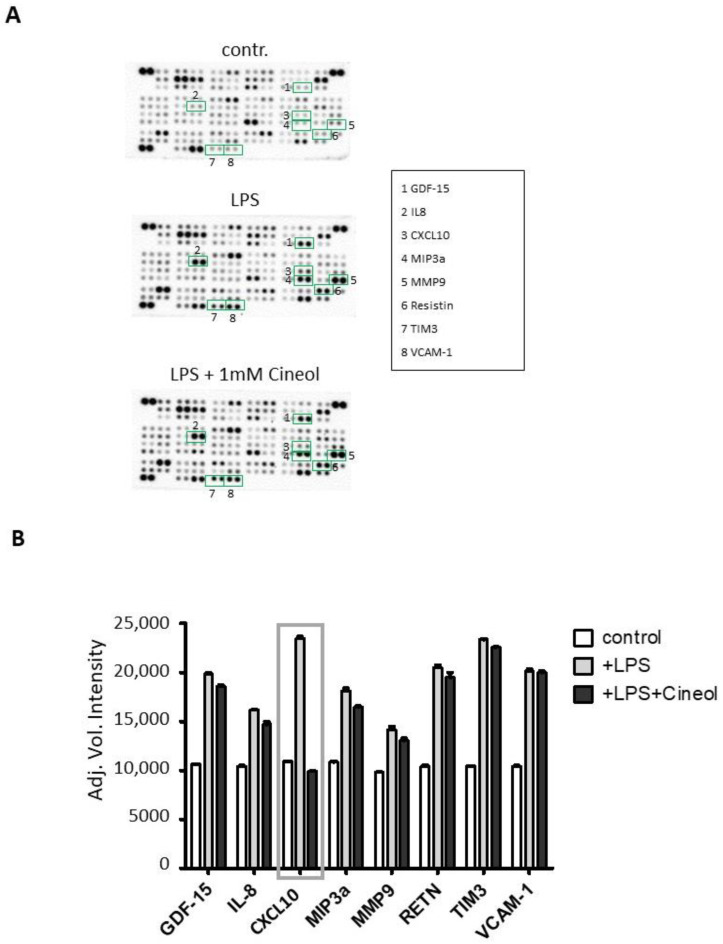
Monocytic cytokines in responses to lipopolysaccharid (LPS) in the absence and presence of 1,8-Cineol. (**A**) Raw images of cytokine arrays of THP-1 cell culture supernatants after 24 h of treatment with LPS and LPS + 1,8-Cineol compared to the medium control. Numbers indicate differential densities of bands of certain cytokines as listed in the box. (**B**) Semiquantitative analysis was performed by measuring the density of the dots and this revealed differential secretion patterns of different cytokines (GDF-15, IL-8, CXCL10, MIP3a, MMP9, RETN, TIM3, VCAM-1) compared to the internal medium control.

Semiquantitative analyses were performed by measuring the density of the dots and these revealed differential secretion patterns of certain cytokines in response to LPS treatment in the presence and absence of 1,8-Cineol compared to the control (Figure 5A). Overall, the data revealed increased secretion patterns of different cytokines (GDF-15, IL-8, MIP3a, MMP9, RETN, TIM3, VCAM-1) in response to LPS stimulation compared to the medium control with 1,8-Cineol having slightly reducing effects (Figure 5). In this context, cytokine CXCL10 is particularly striking, because its increased secretion in response to LPS was found to be strongly attenuated by 1,8-Cineol (Figure 5B).

**Figure 6:**
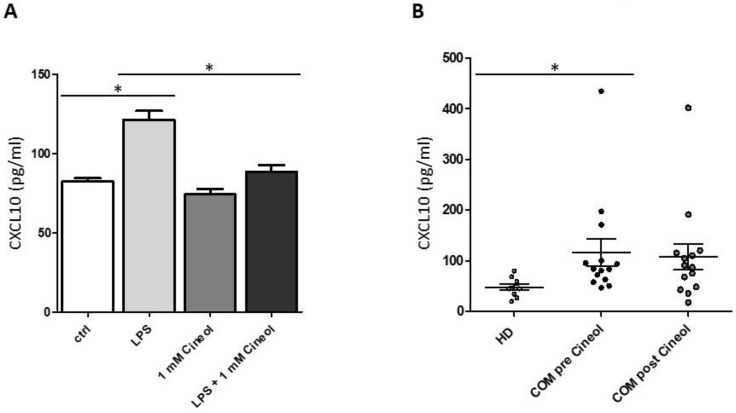
Analysis of CXCL10 concentrations upon 1,8-Cineol treatment. (**A**) ELISA measurements revealed significantly increased CXCL10 secretion levels in THP-1 cell culture supernatants in response to LPS, which significantly decreased upon 1,8-Cineol treatment. (**B**) Significantly increased CXCL10 plasma levels in patients with chronic otitis media (COM; *n* = 14) before 1,8-Cineol administration compared to healthy donors (HD; *n* = 10). *: *p* < 0.05.

To corroborate and quantify these semiquantitative findings, CXCL10 ELISA measurements were performed with THP-1 cell culture supernatants. The data revealed significantly increased CXCL10 secretion levels in response to LPS incubation, which significantly decreased upon 1,8-Cineol treatment of THP-1 monocytes (Figure 6A).

Furthermore, plasma levels of CXCL10 were analyzed in our patient cohort before and after 1,8-Cineol administration and these were compared to healthy donors (Figure 6B). The data revealed heterogeneous but significantly increased levels of plasma CXCL10 in COM patients before 1,8-Cineol administration compared to healthy donors. Post 1,8-Cineol, we could detect a decrease in CXCL10 secretion levels in COM patients (1,8-Cineol-responder), but this was quite heterogeneous and overall not significant between the pre and post 1,8-Cineol situation (Figure 6B). 

## 4. Discussion

### 4.1. Alteration of Monocytic PD-L1 and CX3CR1

We analyzed the impact of the natural plant-derived monoterpene oxide 1,8-Cineol on the cellular characteristics of circulating CD14/CD16 monocyte subsets in the peripheral blood of patients with chronic otitis media (COM) using flow cytometry. In our cohort, we found no significant differences in monocyte subset abundances in COM patients compared to healthy donors and no influence of 1,8-Cineol on the overall subset distribution. Furthermore, we analyzed the expression levels of monocyte adhesion molecules and chemokine receptors CD11a (integrin-α L; LFA-1), CD11b (integrin-α M; Mac-1), CD11c (integrin-α X), and CX3CR1 (CX3CL1 receptor), all of which are involved in the regulation of immune cell binding to endothelial cells and transmigration through the endothelial barrier [1]. CD11b has also been shown to act as an inhibitor of immune functions in myeloid cells [27].

We observed significantly elevated expression levels of all analyzed adhesion molecules in certain monocyte subsets in COM patients, but only CX3CL1 receptor CX3CR1 was found to be significantly down-regulated in intermediate and non-classical CD16^+^ monocytes in response to 14 days of 1,8-Cineol administration. 

Adhesion molecule CX3CR1 enables monocyte crawling along the endothelium and, therefore, is also associated with atherosclerotic plaque development, cardiovascular diseases, and the infiltration of macrophages into inflamed tissues [15,28]. 

Moreover, we have shown significantly increased expression levels of monocytic checkpoint molecule PD-L1 in COM patients, which significantly decreased on intermediate CD16^+^ monocytes upon 14 days of administration of 1,8-Cineol. PD-L1 on monocytes promotes a crucial mechanism for the regulation of T-cell responses preventing overreaction upon inflammation [29,30]. However, an imbalanced activation of the PD-1/PD-L1 pathway can ultimately lead to the development of chronic inflammation and related diseases [31,32]. The possible involvement of the checkpoint molecule PD-L1 (programmed cell death ligand-1) in the development and maintenance of COM has not been intensively elucidated so far. It could only be shown that the PD-1/PD-L1 pathway could be involved in immune exhaustion in response to bovine infections with mycoplasma, a pathogen that causes pneumonia, mastitis, arthritis, and otitis media in cattle [33].

### 4.2. Impact of 1,8-Cineol on LPS-Induced Monocytic Cytokine Secretion

Chronic otitis media is closely associated with distinct inflammatory processes within the middle ear in response to different bacterial and viral infections, as well as with the immunomodulatory response of the middle ear epithelial cells [9,34,35]. The role of CXCL10 in otitis media has hardly been investigated so far. It has recently been shown, in secretory otitis media (SOM), that different inflammatory mediators, such as CXCL1, IL-16, IL-8, IL-17, IL-1β, CXCL10, and CXCL9, were found in high concentrations in middle ear fluids, in association with the presence of bacterial and viral nucleic acid levels [36]. Moreover, it has recently been shown that 1,8-Cineol contributes to a synergistic anti-microbial and also anti-fungal activity within a novel substance based on *Melaleuca alternifolia* leaf oil, which showed inhibitory effects against the pathogenic yeast *Malassezia furfur*, which is involved in the dermatological disease, *Seborrheic dermatitis* [37]. In this context, 1,8-Cineol may be a potential alternative to conventional fungicides, since different fungal citrus pathogens have been shown to develop tolerance to conventional fungicides but not to 1,8-Cineol [38].

In earlier publications, it has already been observed that inflammatory responses in lung macrophages and LPS-induced Egr-1 (expression of early growth response factor-1) expression in THP-1 cells could be suppressed by 1,8-Cineol treatment [39,40]. 

As expected, we detected increased secretion levels of different monocytic inflammatory cytokines in response to LPS stimulation of THP-1 monocytes, whereas cytokine IFN-γ-inducible protein 10 (CXCL10) was particularly interesting because its secretion was found to be strongly attenuated by 1,8-Cineol. Besides its secretion in response to IFN-α, IFN-β or lipopolysaccharide (LPS) [41], the data revealed that elevated circulating IFN-γ and TNF-α synergistically trigger the expression of CXCL10 by podocytes and thus attract activated macrophages to migrate into kidney tissue [42]. It has been shown, in human THP-1 monocytes, that TNF-α acts as a stronger inducer of CXCL10 than IFN-γ in a NF-κB-dependent manner [43]. 

CXCL10 is a chemokine that is abundantly secreted in response to inflammatory stimuli and has been associated with different inflammatory diseases. It has been shown that CXCL10 is required for the secretion of inflammatory cytokines, such as IL-12 and IL-23, by human monocytes, depending on CXCR3 receptor engagement [44]. Moreover, peripheral blood CD14^+^ monocytes, in patients with symptomatic malaria, were identified as the main leucocytic sources of CXCL10. In vitro investigations revealed that monocytes from healthy donors secreted CXCL10 in response to the malaria pathogen, *Plasmodium falciparum* [45]. Elevated CXCL10 expression levels by human monocytes have also been found in patients with *Leishmania braziliensis* infections [46].

It is well established that patients with persisting COM run the risk of developing concomitant diseases such as rheumatoid arthritis. In this context, rheumatoid factor (RF) was detected in 85% of human middle ear effusion and serum samples from patients with chronic otitis media [47]. In this context, it has been indicated that CXCL10 is also associated with disease activity and the perseverance of rheumatoid arthritis [48]. 

The present study revealed new insights into both the bioactive anti-inflammatory effects of 1,8-Cineol and the potential role of cytokine CXCL10 in COM development and maintenance. Our data also revealed significantly elevated levels of plasma CXCL10 in COM patients, but no significant effects of 14 days of 1,8-Cineol administration. This may be due to the investigation period or due to the low aqueous solubility and stability of 1,8-Cineol [49]. As a promising innovative approach to improve the bioavailability of natural plant-derived compounds, the preparation of invasomes (spherical vesicles of phospholipid bilayers) encapsulating thymol, menthol, camphor, and 1,8-Cineol has recently been suggested [50]. An acknowledged limitation of the present study is the relatively small cohort of investigated patients in the analysis of monocyte characteristics. Therefore, further comprehensive investigations on larger patient cohorts over a longer period of time are required to better understand the regulation of systemic immunity in patients with chronic otitis media and to elucidate the interplay with its concomitant diseases such as rheumatoid arthritis.

## Data Availability

The data presented in this study are available on request from the corresponding author.
